# Dark Aberrant Crypt Foci with activated Wnt pathway are related to tumorigenesis in the colon of AOM-treated rat

**DOI:** 10.1186/1756-9966-27-47

**Published:** 2008-09-25

**Authors:** Qing Lu, Bo Jiang, Chen Lin, Tao Shan

**Affiliations:** 1 The Institute for Digestive Medicine, Nanfang Hospital, Southern Medical University, Guangzhou 510515, PR China; 2 Department of Gastroenterology, Guilin Medical College affiliated hospital, Guilin 541001, PR China

In our recent article [1], we did not identify several paragraphs in the background and methods that were reproduced from an article by Paulsen *et al*. [2]. Specifically sections of paragraphs 1 and 2 of the Background were reproduced, as well parts of the sections in the Methods, which describe scoring of classic ACF, dark ACF and tumors, histopathological analysis, and immunohistochemical analysis.

We would also like to acknowledge that what we refer to as Dark ACF in our article is the same as that which Paulsen *et al*. identified as Flat ACF in their article [2].

We apologise to Paulsen *et al. *and regret any inconvenience caused by our failure to acknowledge their work.
